# Ultrasensitive Quantification of Thyroid-Stimulating Hormone and Thyroxine by Nanoelectronic SnS_2_ Transistor Sensors

**DOI:** 10.1021/acssensors.5c00115

**Published:** 2025-06-17

**Authors:** Ankur Anand, Feng-Yi Su, Tse-Hao Chen, Yung-Fu Chen, Yit-Tsong Chen

**Affiliations:** † Department of Electrophysics, 34914 National Yang Ming Chiao Tung University, Hsinchu 300093, Taiwan; ‡ PSMC-NYCU Research Center, National Yang Ming Chiao Tung University, Hsinchu 300093, Taiwan; § LIGHTMED Laser System Research Center, National Yang Ming Chiao Tung University, Hsinchu 300093, Taiwan; ∥ Department of Emergency Medicine, 36897 Mackay Memorial Hospital, Taipei 104, Taiwan; ⊥ Department of Chemistry, 33561 National Taiwan University, Taipei 10617, Taiwan

**Keywords:** thyroid-stimulating hormone, thyroxine, field-effect transistor biosensor, antibody, aptamer, polyethylene glycol, point-of-care diagnostics

## Abstract

The measurement of thyroid hormones in serum is widely regarded as the most valuable single laboratory tool for assessing thyroid function. This study presents a highly sensitive tin disulfide nanosheet-fabricated field-effect transistor (SnS_2_-FET) designed for the detections of human thyroid-stimulating hormone (hTSH) and thyroxine (T4). By co-modifying an antibody (Ab_TSH_ for detecting hTSH), or a DNA aptamer (Apt_T4_ for detecting T4), with polyethylene glycol (PEG) on the SnS_2_-FET channel surface, the PEG:Ab_TSH_/SnS_2_-FET and PEG:Apt_T4_/SnS_2_-FET devices achieve highly sensitive and selective detections of hTSH and T4, respectively, even in a high ionic strength buffer (1× PBS) or undiluted serum. With a low limit of detection (in the femtomolar level) and a wide linear working range (spanning at least 6 orders of magnitude of analyte concentration), the PEG:Ab_TSH_/SnS_2_-FET immunosensor and PEG:Apt_T4_/SnS_2_-FET aptasensor can detect the hTSH and T4 levels encountered in the spectrum of thyroid disorders. Notably, these specific receptor-modified SnS_2_-FET devices display negligible cross-reactivity with other pituitary hormones or serum components. This research indicates that the nanoelectronic SnS_2_-FET sensor platforms hold significant potential for point-of-care clinical diagnostics, particularly for the ultrasensitive detection and early screening of medical conditions.

## Graphical abstract

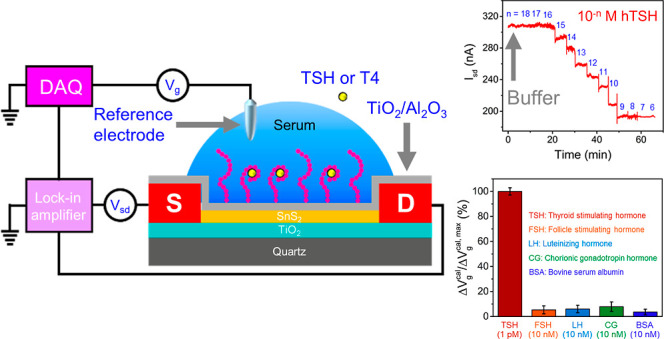

Thyroid disorders, including hypothyroidism, hyperthyroidism, and autoimmune thyroid diseases, are among the most common endocrine disorders globally and affect 5–10% of the population in the world.^1^ The diagnosis and management of these disorders rely heavily on the accurate assessment of thyroid function, which is primarily determined by thyroid function tests through measuring thyroid-stimulating hormone (TSH) and thyroid hormones, specifically thyroxine (T4) and triiodothyronine (T3).^2^ These hormones play crucial roles in regulating metabolism, growth, and development, making their proper functioning vital for overall health. TSH, produced by the pituitary gland, serves as the primary regulator of thyroid hormone synthesis and release. It functions through a feedback mechanism: a low level of thyroid hormones will trigger the release of TSH, which in turn stimulates the thyroid gland to produce more T4 and T3.^3^ Given this regulatory role, TSH is considered to be the most sensitive and specific marker for detecting thyroid dysfunction. An elevated TSH level highly indicates hypothyroidism, while a suppressed TSH level suggests hyperthyroidism, even when thyroid hormones are within the normal range.^2^

In addition to TSH, the direct measurement of thyroid hormones is critical for a comprehensive evaluation of thyroid function. While the TSH level indicates whether the thyroid is functioning properly, thyroid hormone tests provide insight into the severity and specific nature of the disorder.^4^ For example, under subclinical thyroid conditions, the TSH level may be abnormal, but both T4 and T3 remain within normal limits, leading to the necessity of careful interpretation of all hormone measurements for accurate diagnosis and treatment. The interplay between TSH and thyroid hormones forms the foundation of thyroid disorder diagnosis. This relationship is particularly important in conditions like subclinical hypothyroidism, where the TSH level is elevated, but free T4 (fT4) and free T3 (fT3) remain within the reference range.^5^ Similarly, in central hypothyroidism, where the pituitary gland fails to produce adequate TSH, the thyroid hormone levels then become the primary diagnostic indicators.

More than 99% of the circulating T4 and T3 are bound to plasma proteins (e.g., thyroxine-binding globulin, transthyretin, and albumin).^6^ The measurements of total T4 and T3 can be influenced by the factors that alter the protein binding, such as pregnancy, liver disease, nephrotic syndrome, and the use of certain medications (like oral contraceptives or steroids), leading to misleading results.^7^ Although the total T4 and T3 levels change with the variation in thyroxine-binding globulin, the free serum levels of these hormones remain constant. Therefore, fT4 and fT3 provide a more direct assessment of thyroid hormone activities and are particularly valuable in diagnosing thyroid disorders when protein-binding alterations are present.^5^ In particular, an fT4 test is crucial for assessing the thyroid hormone-producing capacity because it provides a direct measurement of the biologically active hormone that influences metabolism.^8^ In contrast, the T3 level may remain within the normal range in the early stage of thyroid dysfunction, particularly in hypothyroidism, making T3 a less reliable marker for initial diagnosis.^2^ The T3 test is often reserved for the cases in which the TSH and T4 results are inconclusive. Therefore, a combination of the TSH and fT4 tests provides a more comprehensive and accurate assessment of thyroid function in clinical practice.^9^ According to the World Health Organization (WHO) guidelines, the normal reference ranges for the TSH and fT4 levels in human serum are 0.4–4.0 mIU/L (i.e., 1.8–18 pM) and 0.8–2.0 ng/dL (i.e., 10.0–26.0 pM), respectively.^2^ The TSH level above this range strongly indicates hypothyroidism; conversely, hyperthyroidism is suggested for the TSH level below this range.

Traditional analytical techniques, such as radioimmunoassay (RIA), chemiluminescence immunoassay (CLIA), and enzyme-linked immunosorbent assay (ELISA), have been the cornerstones of thyroid function tests for decades. Despite their widespread use, these methods come with several limitations, including the need for specialized equipment, trained personnel, and laboratory-based settings, which can hinder timely diagnosis and treatment, especially in resource-limited environments.^10^ Moreover, traditional methods often require invasive blood sampling and are time-consuming, which involve multiple steps from sample collection to result generation.^11^ Recent advancements in biosensing technology offer promising alternatives to traditional diagnostic methods, e.g., electrochemical, optical, and piezoelectric devices, which are designed to provide rapid, real-time monitoring of thyroid hormone levels with minimal invasiveness. However, despite these advancements, contemporary biosensors still face several challenges, such as limited sensitivity,^9^ stability and reproducibility,^12^ and cost and accessibility.^8^ In particular, human TSH (hTSH) is a glycoprotein made up of 204 amino acids and composed of 2 subunits, i.e., the alpha (α) and beta (β) subunits. While the alpha subunit is nearly identical to those of human chorionic gonadotropin (hCG), human luteinizing hormone (hLH), and human follicle-stimulating hormone (hFSH), the beta subunit is unique to hTSH, therefore determining its receptor specificity.^13^ Owing to the structural similarity among these hormones, the development of a biosensor for the specific detection of hTSH has been challenging.

Two-dimensional (2D) materials with a fascinating class of layered structures have garnered significant attention in recent years due to their unique properties and potential applications across various technological domains. 2D semiconductor materials possess several advantages to serve as the conducting channel of a field-effect transistor (FET)-based biosensor, e.g., low sheet resistance, high carrier mobility, large surface area, excellent flexibility and mechanical properties, abundant modification sites, and unique electronic properties. Moreover, 2D materials possess layered structures without dangling bonds, which reduce the scattering centers to maintain a high carrier mobility. Especially, the extremely large surface-to-volume ratio of 2D materials makes all charge carriers flow merely in the thin lamellar structure prone to environmental perturbations, e.g., electric field, bio/chemical molecular bindings, etc., giving rise to ultrasensitive responses to external alterations.^14,15^

Tin disulfide (SnS_2_) is a layered *n*-type semiconductor with a bandgap of 2.1 eV and belongs to the 2D group IV–VI metal dichalcogenide family. SnS_2_ crystallizes in a hexagonal CdI_2_-type lamellar structure with lattice parameters of *a* = 3.74 Å and *c* = 5.94 Å. The SnS_2_ crystal is composed of thin layers stacked upon each other and bound together by van der Waals interaction. Each layer is formed by three atomic planes of S–Sn–S (i.e., one plane of Sn atoms sandwiched between two planes of S atoms), in which the atoms are held together via covalent bonds.^16^ Because of the weak van der Waals interaction between the 2D layers, the SnS_2_ bulk crystal can be mechanically exfoliated to yield nanosheets. The SnS_2_ nanosheets have drawn considerable attention due to the advantages of being low-cost, earth-abundant, nontoxic, and environmentally friendly.^17^ In addition, the SnS_2_ crystal is of good oxidative and thermal stability in air, as well as structural stability in acid and neutral aqueous solutions.^18^ Specifically, SnS_2_ nanosheets-fabricated FET devices (referred to as SnS_2_-FET hereafter) display remarkable electron mobility (230 cm^2^ V^–1^ s^–1^), high transconductance (700 nS), large on–off ratio (>10^6^), and minimal hysteresis,^16^ of which these characteristics make SnS_2_-FET a potentially feasible device for excellent biosensing applications.

Aptamers are engineered single-stranded DNA or RNA sequences isolated through the systematic evolution of ligands by an exponential enrichment (SELEX) technique that possess high affinity and selectivity for their target molecules and have emerged as robust recognition elements in biosensors that rival traditional antibodies.^19,20^ However, their use is often limited by availability, and RNA aptamers, even when available, are susceptible to digestion from serum nucleases. In contrast, a diverse selection of antibodies is readily accessible for various targets, making them facile sources for biosensing applications. Furthermore, antibodies generally exhibit a very high binding affinity to their target molecules, typically in the nano- to picomolar range, making them well-suited for detecting low-abundance biomarkers such as hormones, cytokines, etc. Ito and co-workers isolated a sensitive ssDNA aptamer via the SELEX technique (61-mer, denoted by Apt_T4_) that exhibits strong binding with T4 but almost no affinity to T3.^21^ In addition, the consensus sequence of Apt_T4_ was found to form a stem-loop structure. Based on these considerations, we employed an anti-TSH antibody (represented as Ab_TSH_) and Apt_T4_ for the detection of hTSH and T4, respectively.

In this study, we designed SnS_2_-FET-based biosensor platforms for real-time, ultrasensitive measurements of hTSH and T4, respectively, by modifying the Ab_TSH_ and Apt_T4_ receptors on the SnS_2_-FET surface to capture the corresponding hormone targets. The modification conditions of the receptors on SnS_2_-FET were optimized to cover the TSH and T4 levels found in human serum samples (i.e., ∼1–26 pM), rendering these biosensors eligible for the uses of point-of-care testing.

## Experimental Section

### Device Fabrication of SnS_2_-FET

The SnS_2_-FET devices were fabricated on a cleaned quartz substrate and sandwiched between dielectric layers, comprising a 10 nm thick TiO_2_ bottom layer and a 3/14 nm thick TiO_2_/Al_2_O_3_ top bilayer (as illustrated in Figure 1a). The dielectric layers were deposited by atomic layer deposition (ALD) using tetrakis­(dimethylamido)­titanium (TDMAT) and trimethylaluminum as the precursors for TiO_2_ and Al_2_O_3_, respectively. The TiO_2_ bottom layer, deposited onto a bare quartz substrate, was used to prevent the SnS_2_-FET device from potential interference due to the defects and trap states that could exist on the quartz substrate. After depositing the TiO_2_ bottom layer, mechanically exfoliated SnS_2_ nanosheets were transferred onto the TiO_2_-coated quartz substrate with the aid of polydimethylsiloxane (PDMS) stamping. The SnS_2_-FET devices were fabricated following a standard photolithography process and metallic-electrode deposition on the SnS_2_ nanosheets by sputtering (for Cr) and thermal evaporation (for Au). Finally, the SnS_2_-FET devices were covered with a TiO_2_/Al_2_O_3_ top bilayer to prevent electrical leakage during the biosensing measurements. Detailed procedures to fabricate the SnS_2_-FET devices can be found in Section S2, Supporting Information.

**Figure 1:**
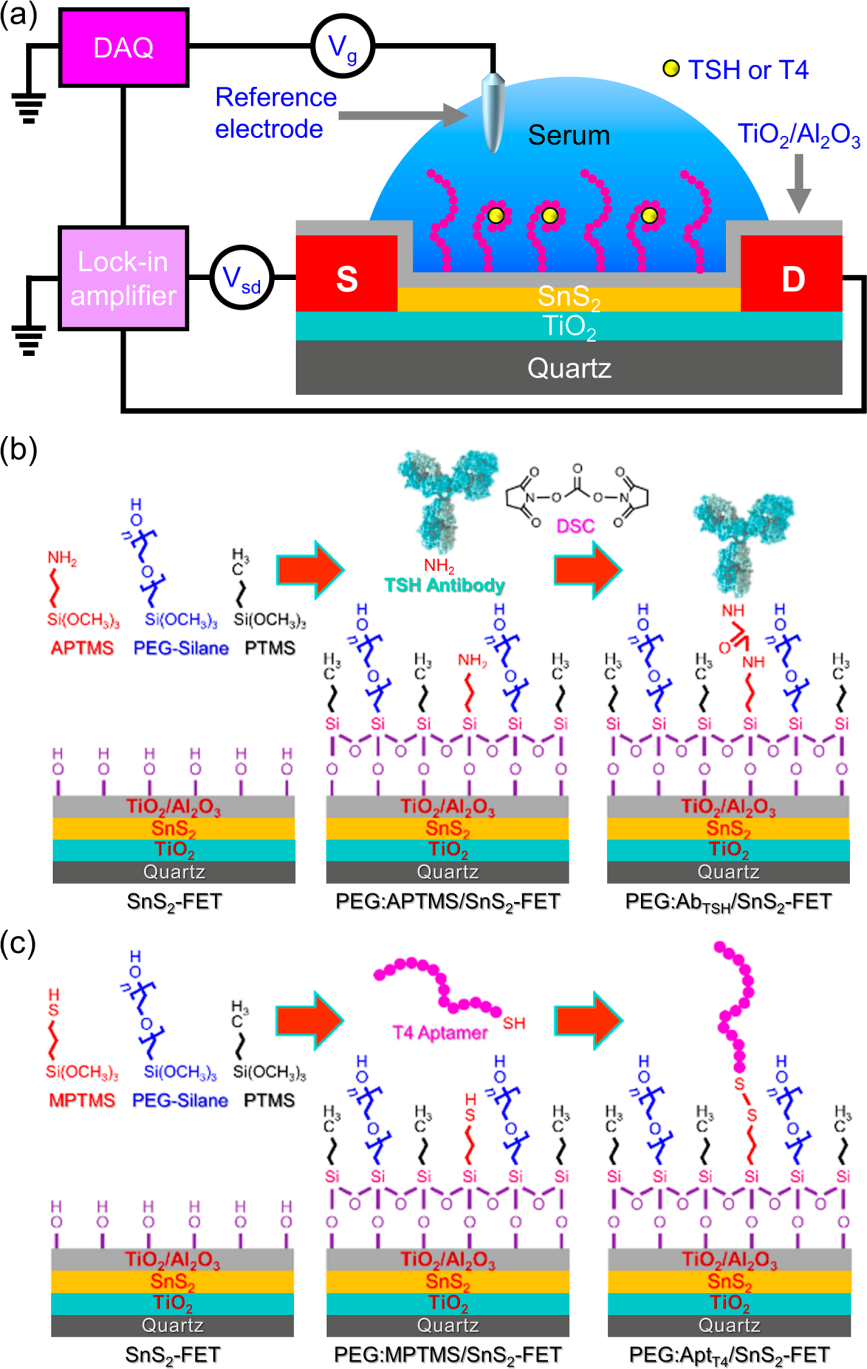
(a) A schematic illustration of the experimental setup of an antibody- or aptamer-modified SnS_2_-FET device for detecting hTSH or T4. (b,c) The chemical modification procedures of immobilizing (b) the anti-TSH antibody and (c) the T4 DNA-aptamer on the SnS_2_-FET surface to form a PEG:Ab_TSH_/SnS_2_-FET immunosensor and a PEG:Apt_T4_/SnS_2_-FET aptasensor, respectively. The drawing is not to scale. Chemicals used in the surface modification include 3-aminopropyl trimethoxysilane (APTMS), 3-mercaptopropyl trimethoxysilane (MPTMS), propyl trimethoxysilane (PTMS), polyethylene glycol (PEG)-silane, and *N*,*N*′-disuccinimidyl carbonate (DSC).

To facilitate delivering the target sample to the SnS_2_-FET surface, a polydimethylsiloxane (PDMS) microfluidic channel with the dimensions of 5 mm (length) × 0.5 mm (width) × 0.5 mm (height) was positioned atop the SnS_2_-FET chip (as shown in Figure S1 of the Supporting Information). Two holes were drilled at the ends of the PDMS channel to allow the insertion of polyethylene tubes (Becton Dickinson, USA, with an inner diameter of 0.38 mm and an outer diameter of 1.09 mm), serving as the inlet and outlet tubes. The inlet tube is connected to the sample reservoir, while the outlet tube is linked to a syringe pump (KD Scientific, USA), which controls the flow of a target solution into the sensor at a predetermined flow rate.

### Surface Modification of SnS_2_-FET

As illustrated in Figure 1a, a TiO_2_/Al_2_O_3_-coated SnS_2_-FET device was subjected to sequential surface modifications to establish the PEG:Ab_TSH_/SnS_2_-FET immunosensor and the PEG:Apt_T4_/SnS_2_-FET aptasensor. For the Ab_TSH_ modification, a 0.1% v/v mixture of 3-aminopropyl trimethoxysilane (APTMS), propyltrimethoxysilane (PTMS), and polyethylene glycol (PEG)-silane (with the concentration ratio of APTMS:PEG:PTMS = 1:2:4) was first incubated for 2 h to facilitate the methoxy groups of APTMS, PTMS, and PEG-silane to react with the hydroxyl groups on the TiO_2_/Al_2_O_3_-coated SnS_2_-FET surface. The device was then heated at 110 °C for 2 h to ensure the formation of a self-assembled monolayer of APTMS, PTMS, and PEG-silane on the TiO_2_/Al_2_O_3_-coated SnS_2_-FET surface via a Ti–O–Si linkage (denoted by PEG:APTMS/SnS_2_-FET in Figure 1b). Subsequently, a solution containing 5 mg of *N*,*N′*-disuccinimidyl carbonate (DSC) in 15 μL of *N*,*N*-diisopropylethylamine (DIPEA) and 1.5 mL of anhydrous dimethyl sulfoxide (DMSO) was dropped on the PEG:APTMS/SnS_2_-FET device for a 2 h reaction. The DSC acts as a cross-linker between the NH_2_ groups of both Ab_TSH_ and APTMS to immobilize Ab_TSH_ on PEG:APTMS/SnS_2_-FET. The device chip was then incubated in an aqueous solution containing Ab_TSH_ for ∼12 h to form the PEG:Ab_TSH_/SnS_2_-FET immunosensor (Figure 1b), followed by washing with phosphate buffered saline (1× PBS, containing 137 mM NaCl, 2.7 mM KCl, 0.5 mM MgCl_2_, 10 mM Na_2_HPO_4_, and 2 mM KH_2_PO_4_, pH 7.4) three times.

For the Apt_T4_ modification, the cleaned TiO_2_/Al_2_O_3_-coated SnS_2_-FET surface was modified with a 0.1% v/v mixture of 3-mercaptopropyl trimethoxysilane (MPTMS), PTMS, and PEG-silane (with the concentration ratio of MPTMS:PEG:PTMS = 1:2:3) for 2 h. Subsequently, the device was heated at 110 °C for 2 h to form a self-assembled monolayer of MPTMS, PTMS, and PEG-silane on the TiO_2_/Al_2_O_3_-coated SnS_2_-FET surface (denoted by PEG:MPTMS/SnS_2_-FET in Figure 1c). The thiol group of MPTMS was utilized for immobilization of the thiol-terminated DNA aptamer (SH-5′-Apt_T4_) via the formation of a disulfide bond. To anchor Apt_T4_, the PEG:MPTMS/SnS_2_-FET surface was first pretreated with 10 mM dithiothreitol (DTT) in 1× PBS at 37 °C for 30 min to cleave the possible disulfide linkage among the MPTMS molecules, rendering free thiol groups available for coupling with the SH-5′-Apt_T4_ aptamers. Thereafter, 10 μM of SH-5′-Apt_T4_ in 2× sodium saline citrate (SSC) buffer was incubated on the PEG:MPTMS/SnS_2_-FET surface, at room temperature for ∼12 h, to construct the PEG:Apt_T4_/SnS_2_-FET aptasensor (Figure 1c) by forming a disulfide bond between SH-5′-Apt_T4_ and MPTMS. Finally, the PEG:Apt_T4_/SnS_2_-FET aptasensor device was washed with 1× PBS three times. The successful immobilizations of Ab_TSH_ and Apt_T4_ on PEG:APTMS/SnS_2_-FET and PEG:MPTMS/SnS_2_-FET, respectively, were confirmed by the transfer-curve measurements and a fluorescence imaging method, as shown in Figure S2. Detailed procedures for the surface modification of SnS_2_-FET devices can be found in Section S3, Supporting Information.

### Spectromicroscopic and Electrical Characterizations

Electron microscopic images were obtained with a JEOL JEM-2100F transmission electron microscope (TEM) operating at 200 kV, which includes an X-Max^*n*^ 100 TSR instrument (Oxford Instruments) for energy-dispersive X-ray spectroscopy (EDS) analysis. An optical microscope (Olympus, BX 51M) equipped with a charge-coupled device (CCD) camera (Leica, DFC495) was employed to examine the size, shape, and color-contrast thickness of mechanically exfoliated SnS_2_ nanosheets. Raman scattering spectra of SnS_2_ nanosheets were collected in a Raman spectrometer (HORIBA JobinYvon) with a 532 nm laser as the excitation source. Electrical measurements in the biosensing experiments were carried out using a lock-in amplifier (Stanford Research Systems, SR 830 DSP), and the solution-gate voltage was supplied by a data acquisition (DAQ, National Instruments) system, controlled with the LabVIEW software.

### Statistical Analysis

All statistical analyses were conducted using OriginLab software. The data are presented in plots and expressed as the mean ± standard deviation.

## Results and Discussion

To build up SnS_2_-FET biosensor platforms, high-quality SnS_2_ nanosheets were exfoliated mechanically from the SnS_2_ bulk crystal. The SnS_2_ bulk crystal was examined by X-ray diffraction (XRD) spectroscopy to possess a hexagonal crystal structure (JCPDS-83-1705, Figure 2a). The Raman scattering spectrum of the mechanically exfoliated SnS_2_ nanosheets (Figure 2b) exhibits a prominent peak at 313.9 cm^–1^, which is ascribed to the A_1g_ phonon mode of SnS_2_. In addition, the Raman signal appearing at 520 cm^–1^ is due to the F_1g_ mode of a Si supporting substrate, which can be used as a frequency reference. The crystallinity of SnS_2_ nanosheets was further characterized by transmission electron microscopy (TEM, Figure 2c) and selected-area electron diffraction (SAED, in the inset of Figure 2c) to confirm a single-crystal hexagonal structure. The chemical compositions of SnS_2_ nanosheets were analyzed by energy-dispersive X-ray spectroscopy (EDS, Figure 2d) with elemental mappings (in the insets of Figure 2d) indicating homogeneous distributions of tin (Sn) and sulfur (S) in the as-prepared SnS_2_ nanosheets.

**Figure 2:**
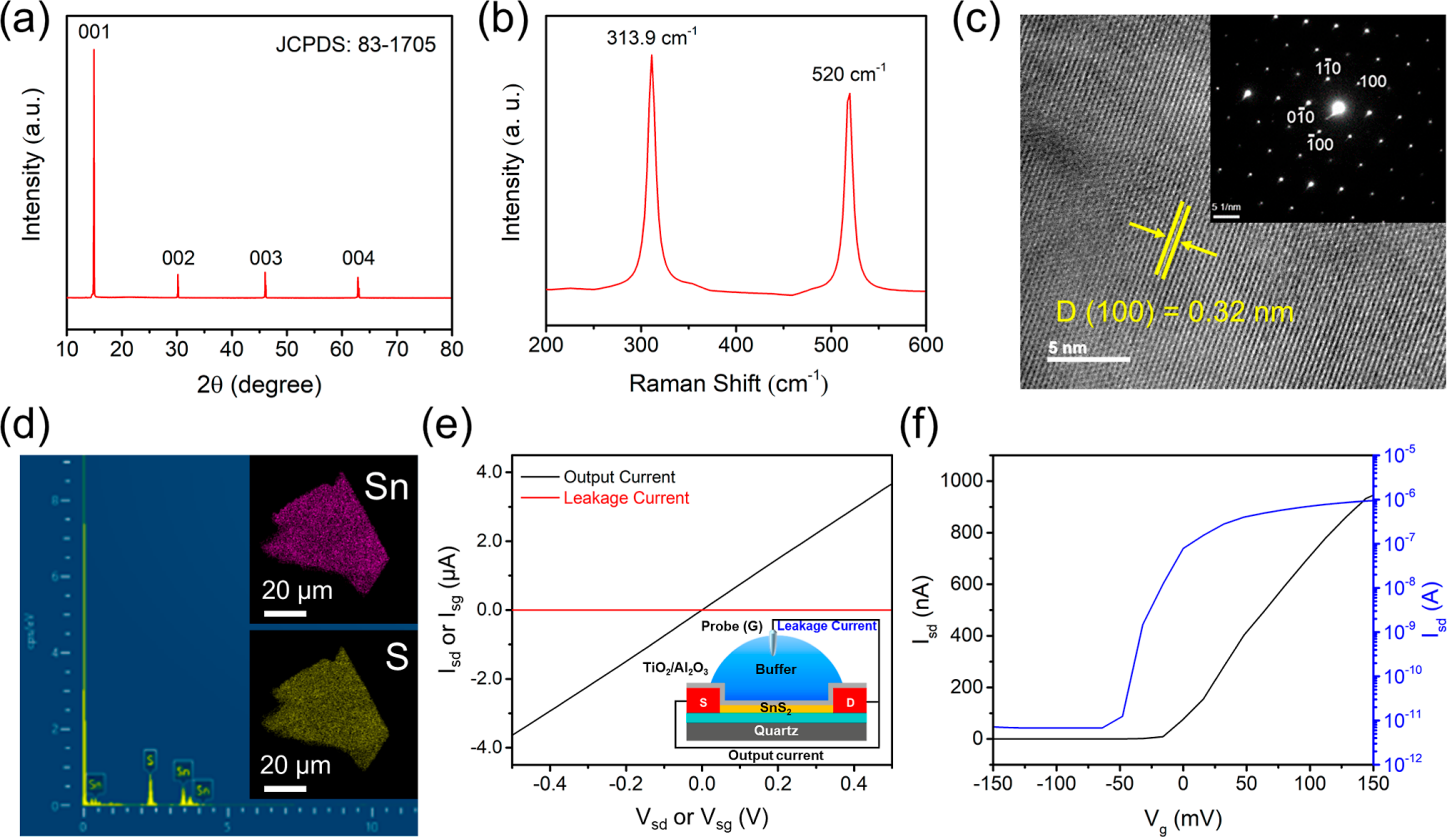
(a) The XRD spectrum of the SnS_2_ bulk crystal reveals a hexagonal crystal structure. (b) The Raman scattering spectrum of mechanically exfoliated SnS_2_ nanosheets exhibits the A_1g_ phonon mode of the SnS_2_ crystal at 313.9 cm^–1^. (c) The TEM image and SAED pattern (in the inset) further indicate the single-crystalline hexagonal structure of SnS_2_ nanosheets. (d) The EDS spectrum and elemental mappings (insets) were obtained to characterize the compositional distributions of the Sn and S atoms in the as-prepared SnS_2_ nanosheets. (e) The output curve (the black *I*_sd_–*V*_sd_ trace) and electrical leakage (the red *I*_sg_–*V*_sg_ trace) of an SnS_2_-FET device were investigated. The electrical leakage of an SnS_2_-FET device was measured, as shown in the inset. (f) The transfer curve (i.e., the *I*_sd_–*V*_g_ plot) of an SnS_2_-FET device, measured by scanning the solution-gate voltage (*V*_g_) with *V*_sd_ = 10 mV, reveals a transconductance of ∼6 μS and an on–off ratio of ∼10^5^.

For the fabrication of SnS_2_-FET devices, the mechanically exfoliated SnS_2_ nanosheets (10–20 nm thick) were transferred onto a TiO_2_ (10 nm thick)-coated quartz substrate, followed by standard photolithographic patterning and electrode (Au/Cr = 50/5 nm thick) deposition. Subsequently, a TiO_2_/Al_2_O_3_ (3/14 nm thick) dielectric bilayer was coated atop the SnS_2_-FET device by atomic layer deposition (ALD), which was used to prevent electrical leakage from the sample solution during SnS_2_-FET biosensing measurements.^22^ Electrical characterizations of the as-fabricated SnS_2_-FETs were examined by the output curve (i.e., the source-drain current (*I*_sd_) vs the source-drain voltage (*V*_sd_) plot, Figure 2e) and transfer curve (i.e., the source-drain current (*I*_sd_) vs the source-gate voltage (*V*_g_) plot, Figure 2f) measurements. The results display that this nanoelectronic device exhibits an ohmic contact (Figure 2e), a high transconductance of typically ∼6 μS measured at *V*_sd_ = 10 mV (the black curve, Figure 2f), and an on–off ratio of ∼10^5^ (the blue curve, Figure 2f). Moreover, at the bias voltage (*V*_sd_ or *V*_sg_) of 0.5 V (Figure 2e), the measured leakage current (*I*_sg_ ∼0.9 nA) is only <0.03% of the channel current (*I*_sd_ ∼3.7 μA), indicating that the TiO_2_/Al_2_O_3_ dielectric bilayer atop the SnS_2_-FET device is an excellent insulator.

As illustrated in Figure 1a, the electrical measurements of an SnS_2_-FET biosensor were conducted with a lock-in amplifier, and the solution-gate voltage was supplied by a data acquisition (DAQ) system through a Ag/AgCl electrode. Details of the device fabrication and electrical measurements of SnS_2_-FET biosensors are delineated in the Experimental Section and Supporting Information. Prior to biosensing measurements, an SnS_2_-FET device involves a series of sequential functionalizations on the channel surface. In this work, we chose Ab_TSH_ or SH-5′-Apt_T4_ as a receptor to be immobilized on SnS_2_-FET for the selective detection of hTSH or T4. To construct an hTSH immunosensor (as illustrated in Figure 1b), an SnS_2_-FET device was first co-modified with a mixture of APTMS, PTMS, and PEG-silane (represented as PEG:APTMS/SnS_2_-FET in Figure 1b). Subsequently, the Ab_TSH_ was immobilized to the amine-terminated APTMS via a cross-linker of DSC to construct a PEG:Ab_TSH_/SnS_2_-FET immunosensor (Figure 1b) for the detection of hTSH. Similarly, a PEG:Apt_T4_/SnS_2_-FET aptasensor was established for the T4 detection (as illustrated in Figure 1c), where an SnS_2_-FET device was first co-modified with a mixture of MPTMS, PTMS, and PEG-silane to form a PEG:MPTMS/SnS_2_-FET (Figure 1c). The SH-5′-Apt_T4_ was then anchored on MPTMS via a disulfide bond to build up a PEG:Apt_T4_/SnS_2_-FET aptasensor (Figure 1c). The disulfide linkage between MPTMS and SH-5′-Apt_T4_ can be chemically reduced by dithiothreitol (DTT) to restore the Apt_T4_ receptor to its native form, making the PEG:Apt_T4_/SnS_2_-FET device reusable.^23,24^

In biosensing measurements with these PEG:Ab_TSH_/SnS_2_-FET and PEG:Apt_T4_/SnS_2_-FET devices, the Debye screening effect to reduce electrical-signal propagation in electrolytic solutions was circumvented by co-modifying the SnS_2_-FET surface with the porous and biomolecule permeable PEG polymer of a high molecular weight (∼10 kDa). This co-modified PEG alters the dielectric constant of the aqueous medium immediately adjacent to the FET surface, thereby increasing the effective Debye screening length (λ_D_) for FET-biosensing measurements to enable the detection of targets in high salt buffers or biological media.^24,25^ The PTMS, on the other hand, was used to adjust the adequate density of antibodies or aptamers, modified on the SnS_2_-FET surface, to ensure enough space for the distribution of Ab_TSH_ or the proper folding of Apt_T4_ when binding with targets. The PTMS also provided a chemically inert SnS_2_-FET surface to prevent nonspecific binding in biosensing measurements.^26,27^ The surface functionalization on a TiO_2_-coated SnS_2_-FET device was examined by (i) transfer-curve measurements (Figure S2a,c) and (ii) a fluorescence imaging method (Figure S2b,d and Section S4) to confirm the successful sequential modifications.

Figure 3a presents the real-time electrical response (i.e., the change in *I*_sd_) of a PEG:Ab_TSH_/SnS_2_-FET immunosensor to different concentrations of hTSH (denoted by *C*_TSH_) in 1× PBS. The sample solution of various *C*_TSH_ was delivered to the immunosensor surface through a PDMS microfluidic channel driven by a syringe pump. To achieve the best detection sensitivity of hTSH by PEG:Ab_TSH_/SnS_2_-FET, an optimal Ab_TSH_:PEG:PTMS = 1:2:4 mixture to be modified on the SnS_2_-FET surface was obtained through a series of tests prior to biosensing detections (as shown in Figure S3a). The isoelectronic point (pI) of hTSH is 6.9;^28^ therefore, in the 1× PBS buffer at pH 7.4, hTSH is negatively charged. In the detection of hTSH, the electrical conductance of the *n*-type PEG:Ab_TSH_/SnS_2_-FET device decreases progressively with increasing *C*_TSH_ due to the electrostatic gating effect. Moreover, the PEG:Ab_TSH_/SnS_2_-FET immunosensor demonstrates a rapid response to hTSH with a response time less than 1 min.

**Figure 3:**
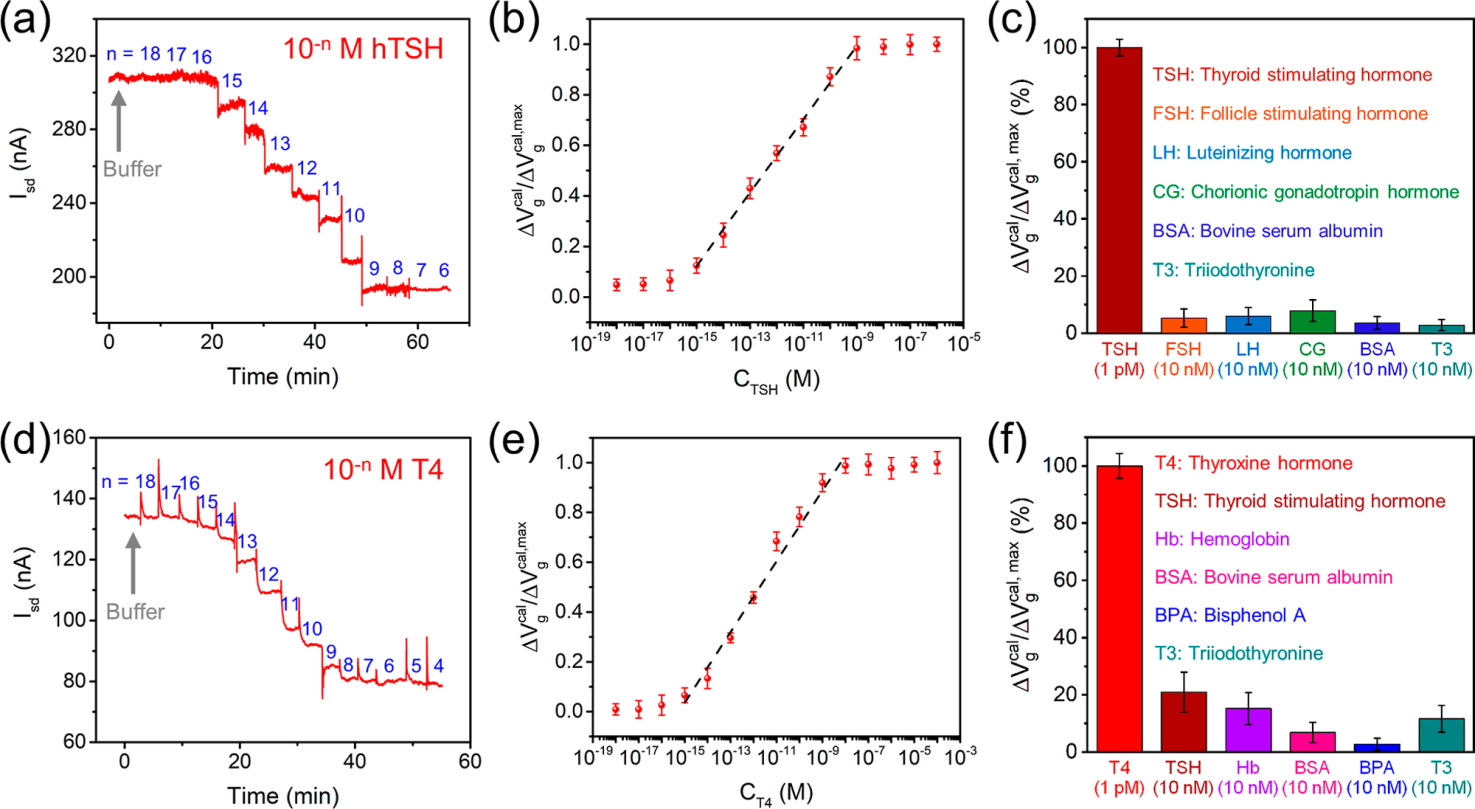
(a) Real-time electrical response and (b) the calibrated response of a PEG:Ab_TSH_/SnS_2_-FET immunosensor to various concentrations of hTSH (*C*_TSH_ = 1 aM to 1 μM) in 1× PBS at pH 7.4. The PEG:Ab_TSH_/SnS_2_-FET device exhibits a very wide probing range with an LWR at *C*_TSH_ = 1 fM to 1 nM and an ultrahigh detection sensitivity with an LOD of ∼1 fM. (c) The target selectivity of a PEG:Ab_TSH_/SnS_2_-FET immunosensor was tested by measuring hTSH (1 pM) against several structural homologues of TSH (10 nM). (d) Real-time electrical response and (e) the calibrated response of a PEG:Apt_T4_/SnS_2_-FET aptasensor to *C*_T4_ = 1 aM to 100 μM in 1× PBS at pH 7.4. The PEG:Apt_T4_/SnS_2_-FET device exhibits an LWR at *C*_T4_ = 1 fM to 10 nM and an LOD of 1 fM. (f) The target selectivity of a PEG:Apt_T4_/SnS_2_-FET aptasensor was tested by measuring T4 (1 pM) against several serum components and Bisphenol A (10 nM). The black dashed lines in (b) and (e) are a guide to the eye, indicating the LWR. Error bars are the mean ± standard deviation with an average from three independent experiments (*n* = 3).

To avoid device-to-device variation in the detection sensitivity with different SnS_2_-FETs, the measured current change due to the receptor-target binding (Δ*I*_sd_ at *V*_g_ = 0 V, relative to the buffer solution as illustrated in Figure S4) was converted to the changes in *V*_g_ (termed the “calibrated response” and represented as Δ*V*_g_^cal^) according to the *I*_sd_–*V*_g_ transfer curve of the SnS_2_-FET device used.^29,30^ In Figure 3b, the data points of the Δ*V*_g_^cal^/Δ*V*_g_^cal,*max*^ vs *C*_TSH_ plot are extracted from those of Figure 3a. Here, Δ*V*_g_^cal,max^ represents the maximum change in the saturated calibrated response of PEG:Ab_TSH_/SnS_2_-FET at *C*_TSH_ ≥ 1 nM. As shown in Figure 3b, the PEG:Ab_TSH_/SnS_2_-FET is highly sensitive for detecting hTSH with a limit of detection (LOD) of 1 fM. The dissociation constant (*K*_d_) of the Ab_TSH_–TSH complex is 3.0 ± 0.2 pM, determined by a least-squares fit of the data points to the Langmuir adsorption isotherm model (Section S7 and Figure S5a). Furthermore, the linear working range (LWR) covers a broad scope of *C*_TSH_ = 1 fM to 1 nM (spanning 6 orders of magnitude of analyte concentration, Figure 3b). This detection range adequately includes the *C*_TSH_ encountered in blood samples of euthyroid, hypothyroid, and hyperthyroid patients. This remarkable achievement in biosensing detections of hTSH with an ultralow LOD and very wide LWR by PEG:Ab_TSH_/SnS_2_-FET stands in stark contrast to several other biosensing techniques reported previously (Table S1). Next, we tested the target selectivity of the PEG:Ab_TSH_/SnS_2_-FET immunosensor against several structural homologues of thyroid hormone. As demonstrated in Figure 3c, the responses of a PEG:Ab_TSH_/SnS_2_-FET against hFSH, hLH, hCG, bovine serum albumin (BSA), and T3 are low, even when they were tested with a much higher concentration (of 10 nM) than hTSH (of 1 pM).

Similarly, Figure 3d,e shows real-time electrical conductance changes and the calibrated response of a PEG:Apt_T4_/SnS_2_-FET aptasensor to different concentrations of T4 (denoted by *C*_T4_) in 1× PBS. Prior to biosensing measurements, a mixture of Apt_T4_:PEG:PTMS with an optimal concentration ratio of 1:2:3 was tested (Figure S3b) and then modified on the SnS_2_-FET surface for the best detection sensitivity of T4. In Figure 3d, since the pI of T4 is ∼7.0,^31^ T4 carries negative charges in 1× PBS at pH 7.4,^32^ making the electrical conductance of the *n*-type PEG:Apt_T4_/SnS_2_-FET device descending as *C*_T4_ increases. As shown in Figure 3e, the PEG:Apt_T4_/SnS_2_-FET aptasensor is highly sensitive for detecting T4 with an LOD of 1 fM and an LWR of 1 fM to 10 nM (spanning 7 orders of magnitude of analyte concentration). This remarkable probing range allows for detecting both fT4 and total T4 found in the blood samples of patients with thyroid disorders. As listed in Table S2, the PEG:Apt_T4_/SnS_2_-FET aptasensor demonstrates greater performance (LOD and LWR) compared to other techniques. The *K*_d_ = 14.4 ± 0.8 pM of the Apt_T4_–T4 complex was obtained by a least-squares fit of the data points to the Langmuir adsorption isotherm model (Section S7 and Figure S5b).

Unlike most biosensing techniques that rely on biomolecule labeling or nanoparticle conjugation to generate or enhance signals, of which the processes can be cumbersome, time-consuming, and may also involve multiple steps, the PEG:Apt_T4_/SnS_2_-FET aptasensor allows for label-free, real-time quantification of T4. This capability is particularly significant for early screening of thyroid disorders. For instance, congenital hypothyroidism is one of the most prevalent inherited metabolic diseases globally and is a leading cause of treatable mental retardation in children. Because the signs and symptoms of this condition are often subtle and not easily recognizable, newborns must undergo screening for the early detection of congenital hypothyroidism at birth. Furthermore, the target selectivity of a PEG:Apt_T4_/SnS_2_-FET was examined against several serum components (TSH, hemoglobin (Hb), BSA, and T3) and Bisphenol A, which is regarded as a common pollutant that can affect the thyroid hormone gene regulation.^33^ The excellent target selectivity of the PEG:Apt_T4_/SnS_2_-FET aptasensor to T4 is evidenced by the tests shown in Figure 3f, in which the signal response to T4 (of 1 pM) is at least several fold higher than the other test objects even with their concentrations (of 10 nM) higher by 4 orders of magnitude. The T3 exhibited a markedly low specificity toward the PEG:Apt_T4_/SnS_2_-FET aptasensor, as negative selection was employed during the isolation of the T4 aptamer to eliminate ss-oligonucleotides that bind to T3.^21^ We also conducted T4 measurements with the same PEG:Apt_T4_/SnS_2_-FET device for three rounds, as shown in Figure S6 and Section S8. The regeneration of the PEG:Apt_T4_/SnS_2_-FET device was performed according to the regeneration protocol provided in Section S3. Notably, the regenerated PEG:Apt_T4_/SnS_2_-FET device exhibited consistent performance (Figure S6) for measuring T4 in 1× PBS in all three rounds of measurements (*N* = 3).

Encouraged by the successful detections of hTSH and T4 in 1× PBS, we sought to demonstrate these detections in hTSH- and T4-depleted serum. After the serum was spiked with various concentrations of hTSH or T4, sensing measurements were conducted to evaluate the response of a PEG:Ab_TSH_/SnS_2_-FET immunosensor or a PEG:Apt_T4_/SnS_2_-FET aptasensor in a clinically relevant whole serum medium. Directly detecting biomarkers in serum will optimize the sensor performance, including specificity, sensitivity, and response time, making it better suited for clinical diagnostics. As presented in Figure 4a, the electrical conductance of a PEG:Ab_TSH_/SnS_2_-FET immunosensor exhibits a dose-dependent decrease with increasing *C*_TSH_. As before, the real-time data are converted to a calibrated response (the Δ*V*_g_^cal^/Δ*V*_g_^cal,max^ vs *C*_TSH_ plot) in Figure 4b, revealing an LOD of 10 fM and an LWR of 10 fM to 10 nM (encompassing 6 orders of magnitude of analyte concentration). The LOD of detecting TSH in serum is 1 order of magnitude inferior to that measured in 1× PBS (i.e., 10 fM vs 1 fM, Figure 4e). This reduced detection sensitivity might be a consequence of the performance in a more complex environment such as serum, in which constituent proteins and ions could interfere with the Ab_TSH_–TSH binding. Nevertheless, the LWR of a PEG:Ab_TSH_/SnS_2_-FET immunosensor operating in serum is sufficiently wide to cover the physiological TSH concentration.

**Figure 4:**
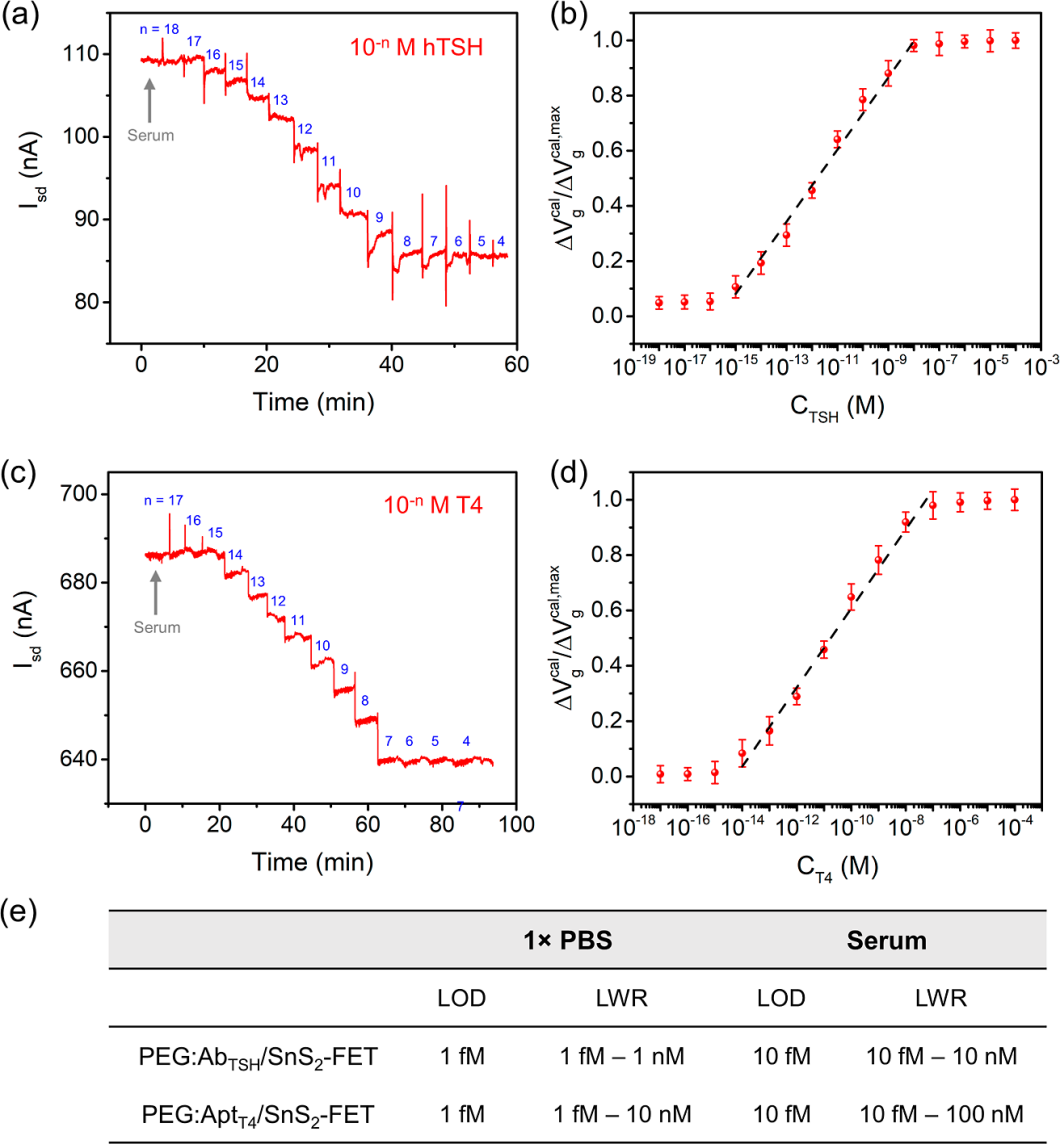
(a) Real-time measurement and (b) the calibrated response of a PEG:Ab_TSH_/SnS_2_-FET immunosensor in the detection of hTSH at *C*_TSH_ = 1 aM to 100 μM in serum at pH 7.4. (c) Real-time measurement and (d) the calibrated response of a PEG:Apt_T4_/SnS_2_-FET aptasensor in the detection of T4 at *C*_T4_ = 10 aM to 100 μM in serum at pH 7.4. (e) A table summarizes the performance metrics for detecting hTSH and T4 in 1× PBS or serum using the PEG:Ab_TSH_/SnS_2_-FET and PEG:Apt_T4_/SnS_2_-FET devices, respectively. The black dashed lines in (b) and (d) are a guide to the eye representing the LWR. Error bars are the mean ± standard deviation with an average from three independent experiments (*n* = 3).

Likewise, Figure 4c shows the real-time response of a PEG:Apt_T4_/SnS_2_-FET aptasensor to the increasing *C*_T4_ in serum, where the electrical conductance of PEG:Apt_T4_/SnS_2_-FET decreases in a concentration-dependent manner. The calibrated response curve in Figure 4d presents an LOD of 10 fM and an LWR of 10 fM to 100 nM. Despite the slightly lower sensitivity of PEG:Apt_T4_/SnS_2_-FET for detecting T4 in serum (LOD of 10 fM), relative to in 1× PBS (LOD of 1 fM, Figure 4e), the probing range still covers the physiological T4 concentration. We also sought to demonstrate that the PEG:Apt_T4_/SnS_2_-FET aptasensor can detect fT4 within the physiological range of euthyroid individuals, i.e., *C*_T4_ = 10.0–26.0 pM. Figure S7a illustrates the real-time sensing of fT4 at *C*_T4_ = 260 fM to 50 pM. The concentration-dependent conductance decline indicates that this aptasensor is capable of detecting fT4 within this specified concentration range. Furthermore, the PEG:Apt_T4_/SnS_2_-FET aptasensor maintained linearity (with the coefficient of correlation *R* ^2^ = 0.997) across the tested concentration range (Figure S7b).

In the field of FET-based biosensors, when using a 3D structure as a conducting channel, the external environment can affect only electrical properties (e.g., carrier density and mobility) of the outer material surface, resulting in low efficiency of transforming into the electrically transduced signals. By contrast, the charge flow of a 2D material-fabricated FET is restricted mainly to the channel surface and is exposed directly to the environment, enabling rapid and sensitive responses to foreign stimuli. On the other hand, for 1D nanowire/nanotube-based FET biosensors, the convex surface of 1D objects with small radii could limit the accessibility of a host of functional groups for surface modification. Comparatively, a 2D material-fabricated FET with the large planar channel area can provide a multitude of modification sites for the uniform chemical functionalization of specific receptors (e.g., Ab_TSH_ and Apt_T4_ in this study). This highly efficient loading of receptors on the 2D conducting channel increases the binding sites drastically for target detection, therefore translating into an extended LWR of detecting receptor–target bindings (as demonstrated in Figures 3 and 4 and compared in Tables S1 and S2). By the same token, a vast number of specific receptors (Ab_TSH_ or Apt_T4_) modified on the large channel surface of a 2D FET device (like the PEG:Ab_TSH_/SnS_2_-FET immunosensor and PEG:Apt_T4_/SnS_2_-FET aptasensor) create a higher probability to capture low-concentration targets (hTSH and T4), commonly driven by the diffusion-limited laminar flow in an ordinary microfluidic channel used in the FET-biosensing measurements,^14,15^ consequently leading to a much better detection sensitivity (as compared in Tables S1 and S2).

## Conclusions

In summary, the label-free and specific detection of hTSH and T4 in whole serum has been successfully demonstrated without the need for sample pretreatment, labeling, or washing steps. To address the Debye screening effect in FET-biosensing measurements, a mixed functionalization layer was developed, incorporating a receptor (Ab_TSH_ or Apt_T4_) along with PEG on the SnS_2_-FET surface. Under these conditions, the PEG:Ab_TSH_/SnS_2_-FET immunosensor and PEG:Apt_T4_/SnS_2_-FET aptasensor exhibit their LODs of the ten femtomolar level and display a wide LWR of at least 6 orders of magnitude of their target concentration. These characteristics give the SnS_2_-FET sensors competitive performance compared with leading technologies. Additionally, these nanoelectronic sensors respond rapidly in less than 1 min, highlighting their potential for early high-throughput screening. This capability is particularly beneficial in critical situations, such as myxedema coma and thyrotoxic crisis, where prompt interventions are necessary to avert life-threatening complications. Importantly, the PEG:Ab_TSH_/SnS_2_-FET immunosensor and PEG:Apt_T4_/SnS_2_-FET aptasensor have demonstrated negligible cross-reactivity with other molecules, ensuring a high selectivity for the hTSH and T4 targets. Overall, the results presented in this work indicate that an SnS_2_-FET-configured biosensor platform, with its tailored surface modifications, holds significant promise for medical diagnostic applications, particularly in point-of-care testing.

## Supplementary Material



## Notes

Materials and reagents, device fabrication of SnS_2_-FET, surface modification of SnS_2_-FET, fluorescence image of a TiO_2_-coated Si substrate, spectromicroscopic and electrical characterizations, preparation of serum samples, microfluidic channel setup, validation of the immobilizations by the transfer-curve measurements and a fluorescence imaging method, optimization of concentration ratios of the Ab_TSH_:PEG:PTMS and Apt_T4_:PEG:PTMS mixtures on the SnS_2_-FET surface, device-to-device variation, Langmuir adsorption isotherm model and dissociation constants of the Ab_TSH_–TSH complex and the Apt_T4_–T4 complex, measurements of T4 using the same PEG:Apt_T4_/SnS_2_-FET device for three rounds, real-time measurement of T4 with a PEG:Apt_T4_/SnS_2_-FET aptasensor within the physiological concentrations, comparison of the detections of thyroid stimulating hormone (TSH) with various biosensing techniques, and comparison of the detections of thyroxine (T4) with various biosensing techniques (PDF)
