# Supplementary material for: Ultrasensitive Quantification of Thyroid-Stimulating Hormone and Thyroxine by Nanoelectronic SnS2 Transistor Sensors
Source: ACS Sens. 2025 Jun 17;10(6):4095–104. doi: 10.1021/acssensors.5c00115 (PMC12210252; doi:10.1021/acssensors.5c00115)
Supplement: Supplementary file 1 [file se5c00115_si_001.pdf]

Supporting Information

Ultrasensitive Quantification of Thyroid-Stimulating Hormone and Thyroxine by Nanoelectronic SnS_2 Transistor Sensors

Ankur Anand,[†,‡,§] Feng-Yi Su,[†] Tse-Hao Chen,[ǁ] Yung-Fu Chen,[†,‡,§]* Yit-Tsong Chen,[†,‡,§, ⊥]*

[†]Department of Electrophysics, National Yang Ming Chiao Tung University, Hsinchu 300093, Taiwan

[‡]PSMC-NYCU Research Center, National Yang Ming Chiao Tung University, Hsinchu 300093, Taiwan

[§]LIGHTMED Laser System Research Center, National Yang Ming Chiao Tung University, Hsinchu 300093, Taiwan

[ǁ]Department of Emergency Medicine, Mackay Memorial Hospital, Taipei 104, Taiwan

[⊥]Department of Chemistry, National Taiwan University, Taipei 10617, Taiwan

*E-mail addresses: yfchen@nycu.edu.tw
*E-mail addresses: ytcchem@ntu.edu.tw

S1. Materials and Reagents

The SnS_2 crystal was obtained from 2D Semiconductors (Arizona, USA). Polydimethylsiloxane (PDMS) was acquired from Sil-More Industrial. The molecular biology grade water (Sigma Aldrich) was used throughout the experiments. The chemicals purchased from Merck include anti-TSH antibody (Ab_{TSH}), human thyroid stimulating hormone (hTSH), human follicle-stimulating hormone (hFSH), human luteinizing hormone (hLH), triiodothyronine (T3), bovine serum albumin (BSA), hemoglobin (Hb), (3-aminopropyl)trimethoxysilane (APTMS), (3-mercaptopropyl)trimethoxysilane (MPTMS), propyltrimethoxysilane (PTMS), dithiothreitol (DTT), phosphate buffered saline (10× PBS), and Bisphenol A. The L-thyroxine (T4) and human chorionic gonadotropin (hCG) were acquired from Thermo Fischer Scientific and MP Biomedicals, respectively. The polyethylene glycol polymer (molecular weight of ~10 kDa) was purchased from Nanocs. The TSH- and T4-depleted serum was obtained from Biochemazone. Thiol-terminated T4 DNA-aptamer (i.e., SH-5′-Apt_{T4}) was synthesized by MDBio (Taipei, Taiwan). The sequence of SH-5′-Apt_{T4} is SH-5′- CGC CGT TGG TGT TCG GTC AGG CTT CCG TGG CAA CGG GGC AAA ATG GTA ATC GCG GGG AAC C -3′

S2. Device Fabrication of SnS_2-FET

The SnS_2-FET devices were fabricated on a quartz substrate. The quartz surface was first washed sequentially with deionized water, ethanol, and acetone and then blown dry with N_2. Subsequently, a 10 nm thick TiO_2 film as a bottom dielectric layer was deposited on the bare quartz substrate by atomic layer deposition (ALD), using tetrakis(dimethylamido)titanium (TDMAT) as a precursor with 38 ALD cycles at 150 °C. This dielectric layer is used to prevent the SnS_2-FET device from potential interference due to the defects and trap states that may exist on the quartz substrate. Next, few-layered SnS_2 nanosheets were peeled off from a bulk

SnS_2 single crystal using a mechanical exfoliation method. These SnS_2 nanosheets were then transferred onto the TiO_2-coated quartz substrate with the aid of PDMS stamping.

The SnS_2-FET devices were fabricated following a standard photolithography process. First, the S1813 photoresist was spin-coated on SnS_2 nanosheets, followed by UV exposure through a photomask and the development of the pattern in a 2.2% tetramethylammonium hydroxide (TMAH) solution for 15 seconds. Next, metallic electrodes were deposited on the SnS_2 nanosheets by sputtering (for Cr) and thermal evaporation (for Au). After fabricating the SnS_2-FET devices, a top dielectric bilayer of TiO_2/Al_2O_3 (3/14 nm in thickness) was deposited atop the as-fabricated SnS_2-FET by ALD. The 14 nm thick Al_2O_3 layer was deposited first, utilizing trimethylaluminum as a precursor for 50 ALD cycles at 150 °C. Subsequently, a 3 nm thick TiO_2 film was coated over the Al_2O_3 layer, using tetrakis(dimethylamido)titanium (TDMAT) as the precursor for 25 ALD cycles at 150 °C. The top TiO_2/Al_2O_3 dielectric bilayer was deposited to insulate the SnS_2-FET device from electrical leakage to the electrolytic solution during the biosensing experiments.

S3. Surface Modification of SnS_2-FET

The SnS_2-FET device functionalization involves a series of sequential modifications on the SnS_2-FET surface. The as-fabricated SnS_2-FET chip was washed with ethanol and acetone and blown dry with N_2 gas. For the anti-TSH antibody (Ab_{TSH})-modification on SnS_2-FET, a mixture of APTMS, PTMS, and PEG-silane was first allowed to incubate for 2 h to facilitate the methoxy groups of APTMS, PTMS, and PEG-silane to react with the hydroxyl groups on the TiO_2/Al_2O_3-coated SnS_2-FET surface. The device was then rinsed with ethanol, blown dry with N_2 gas, and heated at 110 °C for 2 h to ensure the formation of a self-assembled monolayer (SAM) of APTMS, PTMS, and PEG-silane on the TiO_2/Al_2O_3-coated SnS_2-FET surface via a Ti–O–Si linkage (denoted by PEG:APTMS/SnS_2-FET in Figure 1b of the main text).

Subsequently, a solution containing 5 mg of *N*,*N*′-disuccinimidyl carbonate (DSC) in 15 µL of *N*,*N*-diisopropylethylamine (DIPEA) and 1.5 mL of anhydrous dimethyl sulfoxide (DMSO) was dropped on the PEG:APTMS/SnS_2-FET surface for a 2 h reaction. The DSC acts as a cross-linker between the NH_2 groups of both Ab_{TSH} and APTMS to immobilize Ab_{TSH} on PEG:APTMS/SnS_2-FET. Afterward, the device was washed twice with anhydrous DMSO, blown dry with N_2 gas, and incubated in an aqueous solution containing Ab_{TSH} for ~12 h to form the PEG:Ab_{TSH}/SnS_2-FET immunosensor (Figure 1b of the main text). The PEG:Ab_{TSH}/SnS_2-FET device was washed again with deionized water and blown dry with N_2 gas. In the surface modification on PEG:Ab_{TSH}/SnS_2-FET, the optimal concentration ratio of Ab_{TSH}:PEG:PTMS = 1:2:4 was achieved through a series of tests to obtain the best detection sensitivity for hTSH (as demonstrated in Figure S3a).

Similarly, for the Apt_{T4} aptamer modification, the cleaned TiO_2/Al_2O_3-coated SnS_2-FET surface was incubated with a mixture of MPTMS, PTMS, and PEG-silane for 2 h to allow their methoxy groups to react with the hydroxyl groups on the TiO_2/Al_2O_3-coated SnS_2-FET surface. Subsequently, the device was rinsed with ethanol, blown dry with N_2 gas, and heated at 110 °C for 2 h to form a SAM of MPTMS, PTMS, and PEG-silane on the TiO_2/Al_2O_3-coated SnS_2-FET surface via a Ti–O–Si linkage (denoted by PEG:MPTMS/SnS_2-FET in Figure 1c of the main text). The thiol group (SH) of MPTMS was later used to immobilize the thiol-terminated Apt_{T4} (denoted by SH-5′-Apt_{T4}) via the formation of a disulfide bond.

Before immobilizing Apt_{T4}, the PEG:MPTMS/SnS_2-FET surface was pretreated with 10 mM dithiothreitol (DTT) in 1× PBS at 37 °C for 30 min to cleave the possible disulfide linkage among the MPTMS molecules, rendering free thiol groups available for coupling with the SH-5′-Apt_{T4} aptamers. Thereafter, 10 µM of SH-5′-Apt_{T4} in 2× sodium saline citrate (SSC) buffer was incubated on the PEG:MPTMS/SnS_2-FET surface at room temperature for ~12 h to construct the PEG:Apt_{T4}/SnS_2-FET aptasensor (Figure 1c of the main text) via the formation

of a disulfide bond between SH-5′-Apt_{T4} and MPTMS. The PEG:Apt_{T4}/SnS_2-FET device was then washed with 1× PBS three times. While modifying MPTMS, PTMS, and PEG-silane on the TiO_2/Al_2O_3-coated SnS_2-FET surface, the mixing ratio of MPTMS, PTMS, and PEG-silane was optimized (Apt_{T4}:PEG:PTMS = 1:2:3) to achieve the best sensitivity for T4 detection (Figure S3b).

The optimized component ratio on the PEG:Ab_{TSH}/SnS_2-FET or PEG:Apt_{T4}/SnS_2-FET surface offers several advantages: (i) an adequate density of receptors (Ab_{TSH} or Apt_{T4}) to ensure enough space for the distribution of Ab_{TSH}, or the proper folding of Apt_{T4}, when these receptors bind with their targets (hTSH or T4), (ii) a chemically inert surface occupied by PTMS to prevent nonspecific binding in biosensing measurements, (iii) an effective Debye length elongated by PEG, allowing for the detection of targets (hTSH or T4) in high ionic strength solutions (1× PBS or serum). In addition, the PEG:Apt_{T4}/SnS_2-FET aptasensors can be used as a reusable platform due to the disulfide linkage between MPTMS and SH-5′-Apt_{T4}, which can be chemically reduced by DTT to restore the Apt_{T4} receptor to its native form.[S1,S2] The successful immobilizations of Ab_{TSH} and Apt_{T4} on PEG:APTMS/SnS_2-FET and PEG:MPTMS/SnS_2-FET, respectively, were confirmed using both electrical measurements and confocal fluorescence imaging method, as shown in Figure S2.

S4. Fluorescence Image of a TiO_2-Coated Si Substrate

To demonstrate that the Ab_{TSH} and Apt_{T4} receptors could be successfully immobilized on the PEG:APTMS/SnS_2-FET and PEG:MPTMS/SnS_2-FET surfaces, respectively, we used the carboxyfluorescein (known as a fluorescein amidite (FAM) dye)-tagged Ab_{TSH} and Apt_{T4} to show that the FAM-labelled receptors (Ab_{TSH} and Apt_{T4}) could be uniformly immobilized on a triangular hexagonal patterned APTMS- and MPTMS-modified TiO_2 surfaces (which are similar to the surface of a TiO_2/Al_2O_3-coated SnS_2-FET device used in this study). The

chemical modifications of the APTMS- and MPTMS-modified TiO_2 surfaces follow the functionalization procedures delineated in Section S3.

As shown in Figure S2b (and S2d), the homogenous, intense green fluorescence pattern demonstrates the effective, uniform modification of the FAM–Ab_{TSH} (FAM–Apt_{T4}) on the triangular hexagonal patterned APTMS-modified TiO_2 surface (MPTMS-modified TiO_2 surface), in sharp contrast to the dark surrounding where FAM–Ab_{TSH} (FAM–Apt_{T4}) could not be modified directly on the TiO_2 surface.

S5. Spectromicroscopic and Electrical Characterizations

Electron microscopic images were recorded with a JEOL JEM-2100F (200 KV) transmission electron microscope (TEM) supplied with an X-Max^n 100 TSR instrument (Oxford Instruments) for energy dispersive X-ray spectroscopy (EDS) analysis. An optical microscope (Olympus, BX 51M) equipped with a charge-coupled device (CCD) camera (Leica, DFC495) was employed to investigate the thickness (estimated by color contrast), size, and shape of mechanically exfoliated SnS_2 nanosheets. Raman scattering spectra of SnS_2 nanosheets were observed in a Raman spectrometer (HORIBA Jobin Yvon) using a 532 nm laser as the excitation source. Electrical measurements in the biosensing experiments were carried out using a lock-in amplifier (Stanford Research Systems, SR 830 DSP), and the solution-gate voltage was supplied by a data acquisition (DAQ, National Instruments) system, controlled with the LabVIEW program.

S6. Preparation of Serum Samples

The hTSH- and T4-depleted serum was obtained from Biochemazone. The serum medium was spiked to obtain various concentrations of hTSH or T4 through serial dilution with serum as a diluent.

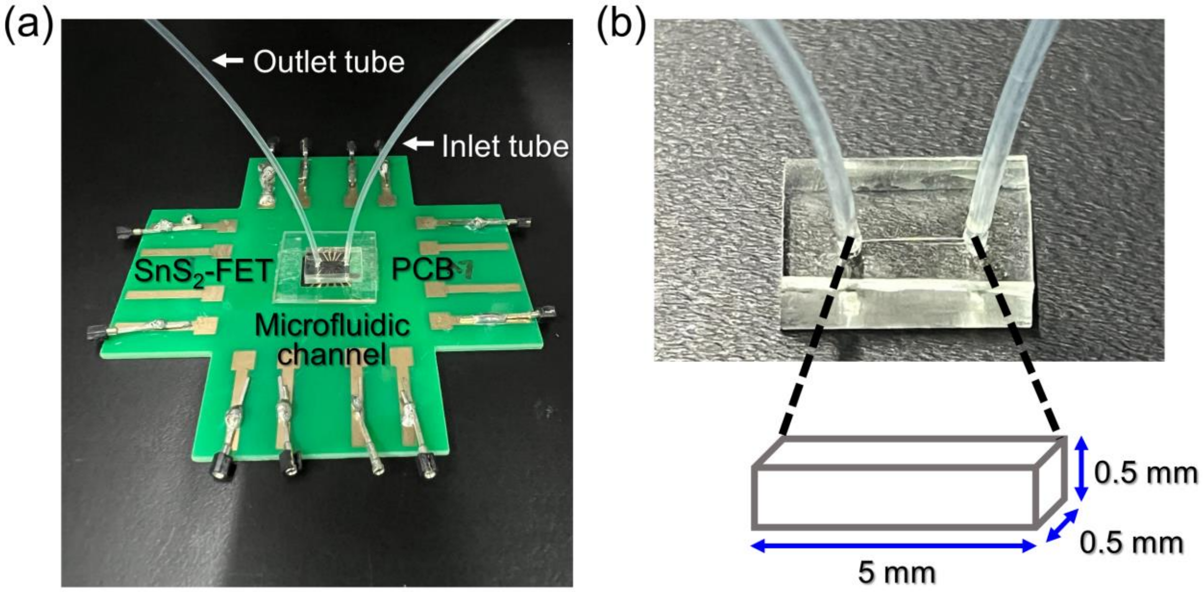

Figure S1. (a) A microfluidic channel mounted on a SnS_2-FET chip. The inlet tube is connected to the sample reservoir, while the outlet tube is linked to a syringe pump. (b) Dimensions of a microfluidic channel at the base of the PDMS cast.

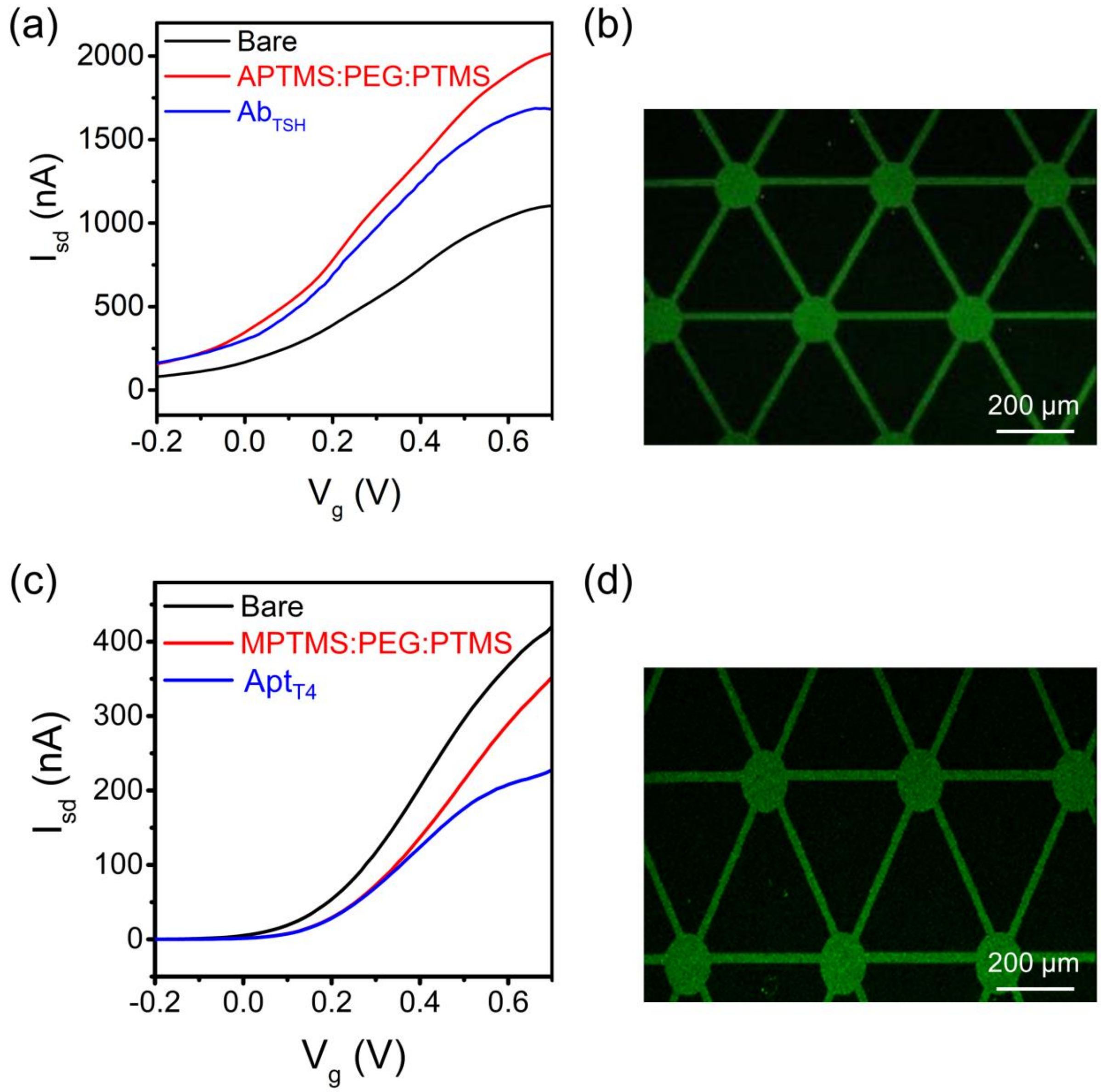

Figure S2. The immobilizations of NH_2-Ab$_{TSH}$-FAM and SH-Apt$_{T4}$-FAM on the TiO_2-coated SnS_2-FET surface are validated by transfer-curve measurements and a fluorescence imaging method. (a) The transfer curves of an SnS_2-FET device were acquired after successive modifications of the APTMS:PEG:PTMS mixture and Ab$_{TSH}$ for the later detection of hTSH. For the measured transfer curves, the increase of the channel current after modifying the APTMS:PEG:PTMS mixture on the TiO_2/Al_2O_3-coated SnS_2-FET surface is caused by the gating effect due to positive charges of the partially protonated NH_2 group of APTMS in 1× PBS at pH 7.4. The decline of the transfer curve after the successive modification of Ab$_{TSH}$ is due to the slightly negatively charged Ab$_{TSH}$ to the *n*-type SnS_2-FET. (b) The green fluorescence from the NH_2-Ab$_{TSH}$-FAM molecules after they were immobilized on a triangular hexagon patterned APTMS-modified TiO_2-coated Si wafer surface has demonstrated the

successful immobilization of NH_2-Ab_{TSH}-FAM on APTMS via the cross-linker of *N*,*N′*-disuccinimidyl carbonate (DSC). The green fluorescence of FAM at 520 nm was excited by a 488 nm radiation source (TCS SP8 X, Leica Microsystems GmbH). (c–d) Similar electrical measurements and fluorescence imaging were applied to demonstrate the successful modification of SH-Apt_{T4}-FAM on the MPTMS-modified surface, except that the chemical bonding between SH-Apt_{T4}-FAM and MPTMS is via the formation of a disulfide bond. The decline of the transfer curve after the successive modifications of the MPTMS:PEG:PTMS mixture and Apt_{T4} is due to the negative charges carried by the thiol group of MPTMS and the phosphate backbone of Apt_{T4} in 1× PBS at pH 7.4.

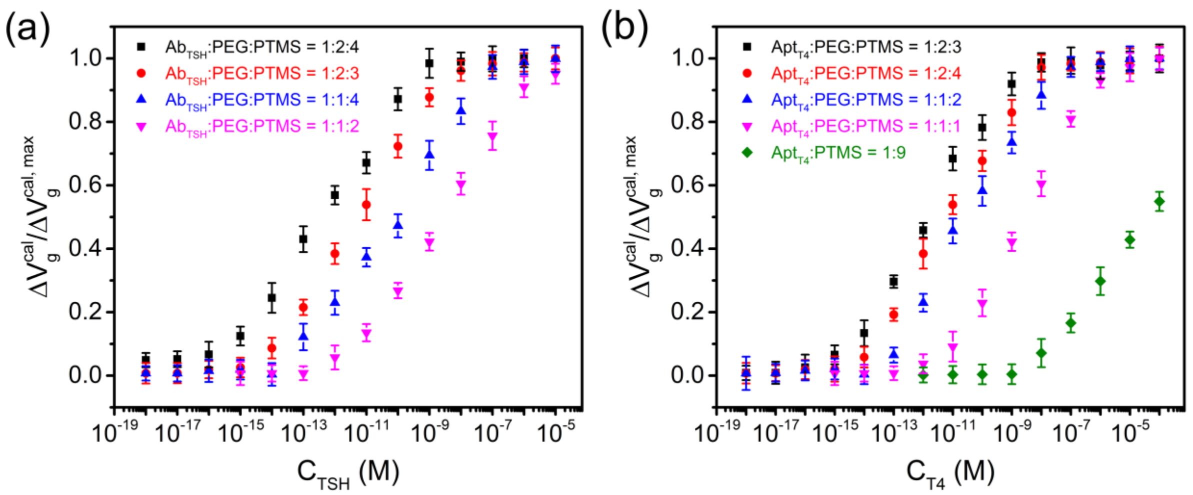

Figure S3. Different concentration ratios of the (a) Ab$_{TSH}$:PEG:PTMS and (b) Apt$_{T4}$:PEG:PTMS mixtures were modified on the SnS_2-FET surface for detecting hTSH and T4, respectively. The optimal ratio of the mixture was obtained by selecting the test with the lowest LOD. Error bars are the mean ± standard deviation with an average from three independent experiments (*n* = 3).

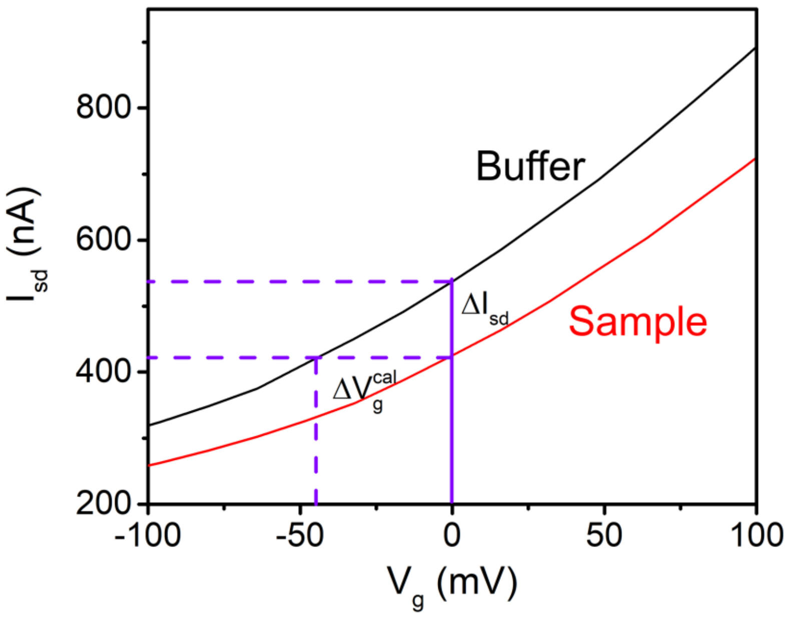

Figure S4. To avoid device-to-device variation in the detection sensitivity with different SnS_2-FETs, the measured current change due to the receptor-target binding (ΔI_{sd} at V_g = 0 mV, relative to the buffer solution) was converted to the changes in V_g (termed the "calibrated response" and represented as ΔV_g^{cal}) according to the I_{sd}–V_g transfer curve of the SnS_2-FET device used.

S7. Langmuir Adsorption Isotherm Model and Dissociation Constant

The dissociation constant (K_d) of the receptor-target complex (Ab_{TSH}–TSH and Apt_{T4}–T4) was determined by a least-squares fit of the data points to the Langmuir adsorption isotherm model.[S2,S3] The data points were extracted from Figure 3b of the main text (for the Ab_{TSH}–TSH complex) and Figure 3e of the main text (for the Apt_{T4}–T4 complex) and plotted as $C_{TSH}/\Delta V^{cal}_{g,\,TSH}$ vs C_{TSH} in Figure S5a (or $C_{T4}/\Delta V^{cal}_{g,\,T4}$ vs C_{T4} in Figure S5b) to determine the K_d of the receptor–target complex. The Langmuir adsorption isotherm model can be expressed as

$$\frac{C_{Target}}{\Delta V^{cal}_{g,\,Target}} = \frac{1}{\Delta V^{cal,\,max}_{g,\,Target}} \cdot C_{Target} + \frac{1}{\Delta V^{cal,\,max}_{g,\,Target}} \cdot K_d \qquad \text{(S1)}$$

where $\Delta V^{cal}_{g,\,Target}/\Delta V^{cal,\,max}_{g,\,Target}$ (%) = $(\Delta V^{cal}_{g,\,Target} - \Delta V^{cal}_{g,\,Buffer})/(\Delta V^{cal,\,max}_{g,\,Target} - \Delta V^{cal}_{g,\,Buffer}) \times 100$ (%), $\Delta V^{cal}_{g,\,Buffer}$ is the calibrated response measured at $C_{Target} = 0$ M, and $\Delta V^{cal,\,max}_{g,\,Target}$ is the saturated calibrated response measured at high C_{Target}.

According to Equation S1, the Y-intercept of the resultant plot (in Figure S5) provides a value of $K_d/\Delta V^{cal,\,max}_{g,\,Target}$, while the slope of the fitted curve yields a value of $1/\Delta V^{cal,\,max}_{g,\,Target}$. Therefore, K_d is determined by dividing the Y-intercept value by the slope value.

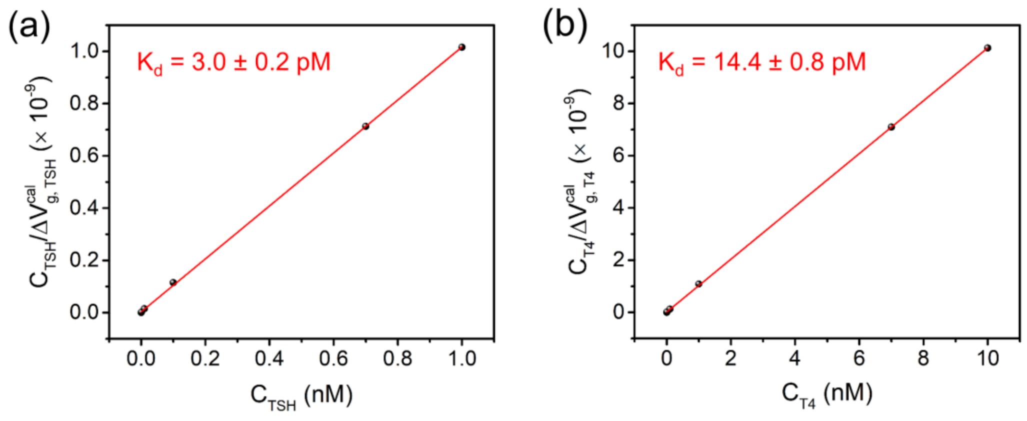

Figure S5. The dissociation constants of (a) the Ab_{TSH}–TSH complex and (b) the Apt_{T4}–T4 complex were determined from the biosensing measurements by PEG:Ab_{TSH}/SnS_2-FET (Figure 3b of the main text) and PEG:Apt_{T4}/SnS_2-FET (Figure 3e of the main text). The

dissociation constants of 3.0 ± 0.2 pM for the Ab_{TSH}–TSH complex and 14.4 ± 0.8 pM for the Apt_{T4}–T4 complex are obtained by a least-squares fit of the data points to the Langmuir adsorption isotherm model.

S8. T4 measurements with a regenerated PEG:Apt_{T4}/SnS_2-FET device

We also obtained the T4 measurements with the same PEG:Apt_{T4}/SnS_2-FET device for three rounds. The regeneration of a PEG:Apt_{T4}/SnS_2-FET device was performed according to the regeneration protocol provided in Section S3. Typically, a PEG:Apt_{T4}/SnS_2-FET device can be repeatedly used three times for electrical measurements before device degradation. After regeneration, the electrical characteristics of the PEG:Apt_{T4}/SnS_2-FET device were restored to their original qualities, as judged by measuring the transfer curves after each round of regeneration.

Figure S6a shows the four transfer curves: one from the original PEG:Apt_{T4}/SnS_2-FET device (black curve) and three obtained after implementing the aforementioned regeneration protocol over three successive rounds. Notably, the transfer curve of the original device nearly coincides with those obtained after the first (red curve) and second (blue curve) rounds of regeneration, indicating that the electrical characteristics of the device remain largely unchanged. In contrast, the transfer curve acquired from the regenerated device after the third round (pink curve) of measurements exhibits noticeable degradation and pronounced hysteresis.

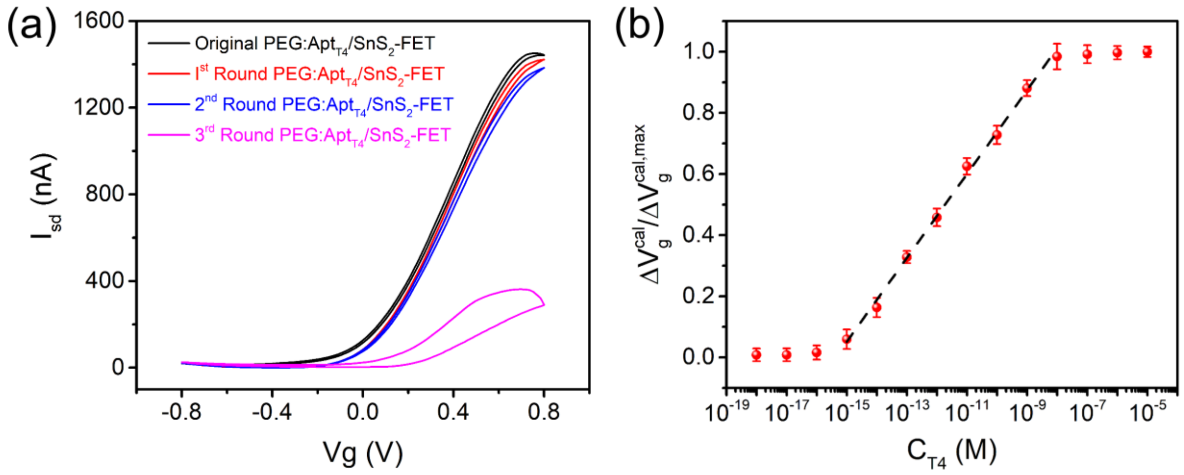

Figure S6. (a) The transfer curves of a PEG:Apt_{T4}/SnS_2-FET device include the original device (black curve) and after the first (red curve), second (blue curve), and third (pink curve) rounds

of regeneration. (b) The calibrated response of a PEG:Apt_{T4}/SnS_2-FET aptasensor for detecting T4 at C_{T4} = 1 aM–10 μM in 1× PBS at pH 7.4. The data is expressed as the mean ± standard deviation obtained from three rounds (N = 3) of T4 sensing using the same regenerated PEG:Apt_{T4}/SnS_2-FET aptasensor.

In general, a PEG:Apt_{T4}/SnS_2-FET device can be regenerated three times for detecting T4 in 1× PBS. Figure S6b presents the calibrated responses of a PEG:Apt_{T4}/SnS_2-FET aptasensor in the detection of T4, at C_{T4} = 1 aM–10 μM in 1× PBS at pH 7.4, using the same regenerated device for three times.

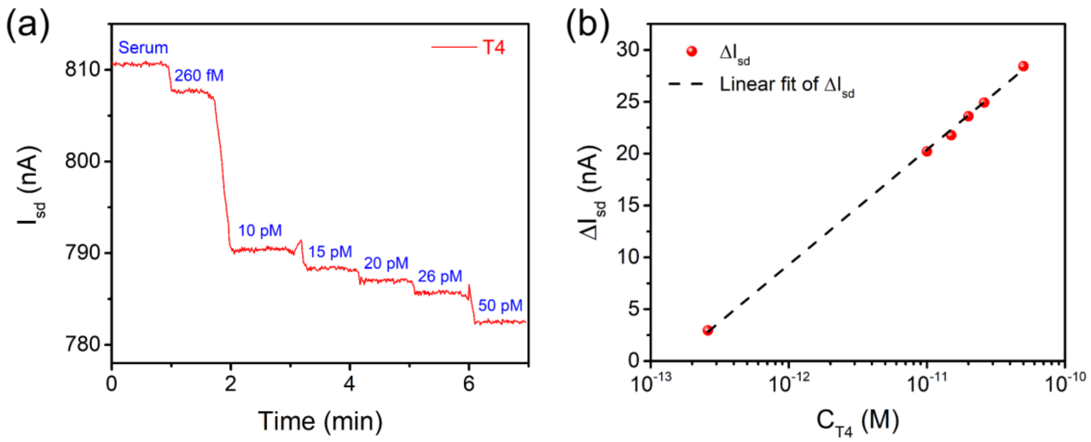

Figure S7. (a) Real-time measurement with a PEG:Apt$_{T4}$/SnS_2-FET aptasensor in the detection of T$_4$ (C_{T4} = 260 fM–50 pM) in serum at pH 7.4. (b) The ΔI_{sd} vs C_{T4} plot shows the linear response of a PEG:Apt$_{T4}$/SnS_2-FET aptasensor to the free T4 concentrations within this physiological range.

Table S1. Comparison of the detections of thyroid stimulating hormone (TSH) with various biosensing techniques

Technique	Sample	Detection Limit	Linear Working Range	Reference
Electrochemical	0.1× PBS buffer	3.57 pM	7.14 pM – 3.21 nM	S4
Electrochemical	0.01 M PBS	5.6 fM	5.6 fM – 5.36 nM	S5
Electrochemical	0.01 M PBS	7.84 pM	7.84 pM – 98 pM	S6
Colorimetric	Buffer	900 fM	900 fM – 53.2 pM	S7
Surface Plasmon Resonance	Serum	129 pM	129 pM – 12 nM	S8
Surface Plasmon Resonance	Water	2.24 pM	2.24 pM – 70 pM	S9
Chemiluminescence	PBS	61.6 fM	2.07 pM – 100.8 pM	S10
Electrochemiluminescence	0.2 M PBS	112 fM	0.28 pM – 112 pM	S11
Gold extended gate FET	Serum	500 fM	500 fM – 10 nM	S12
Graphene-FET	Serum	10 fM	10 fM – 1 nM	S13
Commercial Immmunoassay	Serum	14 fM	14 fM – 560 pM	S14
PEG:Ab_{TSH}/SnS_2-FET	1× PBS buffer	1 fM	1 fM – 1 nM	Present work
PEG:Ab_{TSH}/SnS_2-FET	Serum	10 fM	10 fM – 10 nM	Present work

Table S2. Comparison of the detections of thyroxine (T4) with various biosensing techniques

Technique	Sample	Detection Limit	Linear Working Range	Reference
Electrochemical	Serum	12.9 nM	12.9 nM – 645 nM	S15
Electrochemical	PBS	0.26 fM	0.92 fM – 1.48 pM	S16
Electrochemical	Serum	11.41 pM	1.5 pM – 14.7 nM	S17
Electrochemical	Acetate buffer	0.26 pM	0.64 pM – 645 pM	S18
Electrochemical	0.1 M PBS	0.5 pM	10 pM – 10 nM	S19
Electrochemical	0.1 M PBS	19.35 fM	64.5 fM – 6.45 nM	S20
Surface Plasmon Resonance	PBS	11.75 pM	0.67 pM – 83.98 pM	S21
Fluorescence	Urine	0.47 nM	0 – 500 nM	S22
Chemiluminescence	10 mM PBS	2.2 nM	5 nM – 250 nM	S23
Optical	PBS	17.5 nM	1 nM – 100 nM	S24
PEG:Apt_{T4}/SnS_2-FET	1× PBS buffer	1 fM	1 fM – 10 nM	Present work
PEG:Apt_{T4}/SnS_2-FET	Serum	10 fM	10 fM – 100 nM	Present work

References

(S1) Chiang, P.-L.; Chou, T.-C.; Wu, T.-H.; Li, C.-C.; Liao, C.-D.; Lin, J.-Y.; Tsai, M.-H.; Tsai, C.-C.; Sun, C.-J.; Wang, C.-H.; Fang, J.-M.; Chen, Y.-T. Nanowire Transistor-Based Ultrasensitive Virus Detection with Reversible Surface Functionalization. *Chem.—Asian J.* **2012**, *7*, 2073–2079.

(S2) Anand, A.; Chen, C.-Y.; Chen, T.-H.; Liu, Y.-C.; Sheu, S.-Y.; Chen, Y.-T. Detecting Glycated Hemoglobin in Human Blood Samples Using a Transistor-Based Nanoelectronic Aptasensor. *Nano Today* **2021**, *41*, 101294.

(S3) Li, B.-R.; Hsieh, Y.-J.; Chen, Y.-X.; Chung, Y.-T.; Pan, C.-Y.; Chen, Y.-T. An Ultrasensitive Nanowire-Transistor Biosensor for Detecting Dopamine Release from Living PC12 Cells under Hypoxic Stimulation. *J. Am. Chem. Soc.* **2013**, *135*, 16034–16037.

(S4) Beitollahi, H.; Ivari, S. G.; Torkzadeh-Mahani, M. Application of Antibody–Nanogold–Ionic Liquid–Carbon Paste Electrode for Sensitive Electrochemical Immunoassay of Thyroid-Stimulating Hormone. *Biosens. Bioelectron.* **2018**, *110*, 97–102.

(S5) Saxena, R.; Srivastava, S. A. Sensitive and One-Step Quantification of Thyroid Stimulating Hormone Using Nanobiosensor. *Mater. Today: Proc.* **2019**, *18*, 1351–1357.

(S6) Lin, Z.-H.; Shen, G.-L.; Miao, Q.; Yu, R.-Q. *Anal. Chim. Acta* **1996**, *325*, 87–92.

(S7) Bikkarolla, S. K.; McNamee, S. E.; Vance, P.; McLaughlin, J. High-Sensitive Detection and Quantitative Analysis of Thyroid-Stimulating Hormone Using Gold-Nanoshell-Based Lateral Flow Immunoassay Device. *Biosensors* **2022**, *12*, 182.

(S8) Treviño, J.; Calle, A.; Rodríguez-Frade, J. M.; Mellado, M.; Lechuga, L. M. Surface Plasmon Resonance Immunoassay Analysis of Pituitary Hormones in Urine and Serum Samples. *Clin. Chim. Acta* **2009**, *403*, 56–62.

(S9) Salahvarzi, A.; Mahani, M.; Torkzadeh-Mahani, M.; Alizadeh, R. Localized Surface Plasmon Resonance Based Gold Nanobiosensor: Determination of Thyroid Stimulating Hormone. *Anal. Biochem.* **2017**, *516*, 1–5.

(S10) Shim, C.; Chong, R.; Lee, J. H. Enzyme-Free Chemiluminescence Immunoassay for the Determination of Thyroid Stimulating Hormone. *Talanta* **2017**, *171*, 229–235.

(S11) Liu, Y.; Zhang, Q.; Wang, H.; Yuan, Y.; Chai, Y.; Yuan, R. An Electrochemiluminescence Immunosensor for Thyroid Stimulating Hormone Based on Polyamidoamine-Norfloxacin Functionalized Pd–Au Core–Shell Hexoctahedrons as Signal Enhancers. *Biosens. Bioelectron.* **2015**, *71*, 164–170.

(S12) Gutiérrez-Sanz, Ó.; Andoy, N. M.; Filipiak, M. S.; Haustein, N.; Tarasov, A. Direct, Label-Free, and Rapid Transistor-Based Immunodetection in Whole Serum. *ACS Sens.* **2017**, *2*, 1278–1286.

(S13) Andoy, N. M.; Filipiak, M. S.; Vetter, D.; Gutiérrez-Sanz, Ó.; Tarasov, A. Graphene-Based Electronic Immunosensor with Femtomolar Detection Limit in Whole Serum. *Adv. Mater. Technol.* **2018**, *3*, 1800186.

(S14) Sarkar, R. TSH Comparison between Chemiluminescence (Architect) and Electrochemiluminescence (Cobas) Immunoassays: An Indian Population Perspective. *Indian J. Clin. Biochem.* **2013**, *29*, 189–195.

(S15) Kim, H.-U.; Kim, H. Y.; Seok, H.; Kanade, V.; Yoo, H.; Park, K.-Y.; Lee, J.-H.; Lee, M.-H.; Kim, T. Flexible MoS2–Polyimide Electrode for Electrochemical Biosensors and Their Applications for the Highly Sensitive Quantification of Endocrine Hormones: PTH, T3, and T4. *Anal. Chem.* **2022**, *92*, 6327–6333.

(S16) Zhang, Q.; Chen, X.; Tu, F.; Yao, C. Ultrasensitive Enzyme-Free Electrochemical Immunoassay for Free Thyroxine Based on Three Dimensionally Ordered Macroporous Chitosan–Au Nanoparticles Hybrid Film. *Biosens. Bioelectron.* **2014**, *59*, 377–383.

(S17) Park, S. Y.; Kim, J.; Yim, G.; Jang, H.; Lee, Y.; Kim, S. M.; Park, C.; Lee, M.-H.; Lee, T. Fabrication of Electrochemical Biosensor Composed of Multi-Functional DNA/Rhodium Nanoplate Heterolayer for Thyroxine Detection in Clinical Sample. *Colloids Surf., B* **2020**, *195*, 111240.

(S18) Karami, P.; Gholamin, D.; Johari-Ahar, M. Electrochemical Immunoassay for One-Pot Detection of Thyroxin (T4) and Thyroid-Stimulating Hormone (TSH) Using Magnetic and Janus Nanoparticles. *Anal. Bioanal. Chem.* **2023**, *415*, 4741–4751.

(S19) Kashefi-Kheyrabadi, L.; Koyappayil, A.; Kim, T.; Cheon, Y.-P.; Lee, M.-H. A MoS_2@$Ti_3C_2T_x$ MXene Hybrid-based Electrochemical Aptasensor (MEA) for Sensitive and Rapid Detection of Thyroxine. *Bioelectrochemistry* **2021**, *137*, 107674.

(S20) Han, J.; Zhuo, Y.; Chai, Y.; Yu, Y.; Liao, N.; Yuan, R. Electrochemical Immunoassay for Thyroxine Detection Using Cascade Catalysis as Signal Amplified Enhancer and Multi-Functionalized Magnetic Graphene Sphere as Signal Tag. *Anal. Chim. Acta* **2013**, *790*, 24–30.

(S21) Mradula; Raj, R.; Devi, S.; Mishra, S. Antibody-labeled Gold Nanoparticles Based Immunosensor for the Detection of Thyroxine Hormone. *Anal. Sci.* **2020**, *36*, 799–806.

(S22) Seo, Y. H.; Baik, S.; Lee, J. Nanopore Surface Engineering of Molecular Imprinted Mesoporous Organosilica for Rapid and Selective Detection of L-Thyroxine. *Colloids Surf., B* **2024**, *234*, 113711.

(S23) Huang, Y.; Zhao, S.; Shi, M.; Liu, Y.-M. Chemiluminescent Immunoassay of Thyroxine Enhanced by Microchip Electrophoresis. *Anal. Biochem.* **2010**, *399*, 72–77.

(S24) Smolinska-Kempisty, K.; Guerreiro, A.; Canfarotta, F.; Cáceres, C.; Whitcombe, M. J.; Piletsky, S. A Comparison of the Performance of Molecularly Imprinted Polymer Nanoparticles for Small Molecule Targets and Antibodies in the ELISA Format. *Sci. Rep.* **2016**, *6*, 37638.
